# Astroglial networking contributes to neurometabolic coupling

**DOI:** 10.3389/fnene.2013.00004

**Published:** 2013-04-26

**Authors:** Carole Escartin, Nathalie Rouach

**Affiliations:** ^1^CEA DSV I2BM MIRCen and CNRS URA2210, Fontenay-aux-RosesParis, France; ^2^Neuroglial Interactions in Cerebral Physiopathology, Center for Interdisciplinary Research in Biology, CNRS UMR 7241, INSERM U1050, Collège de FranceParis, France

**Keywords:** astrocytes, neuroglial interactions, gap junctions, astroglial networks, energy metabolism, neurometabolic coupling, neurodegenerative diseases, epilepsy

## Abstract

The strategic position of astrocytic processes between blood capillaries and neurons, provided the early insight that astrocytes play a key role in supplying energy substrates to neurons in an activity-dependent manner. The central role of astrocytes in neurometabolic coupling has been first established at the level of single cell. Since then, exciting recent work based on cellular imaging and electrophysiological recordings has provided new mechanistic insights into this phenomenon, revealing the crucial role of gap junction (GJ)-mediated networks of astrocytes. Indeed, astrocytes define the local availability of energy substrates by regulating blood flow. Subsequently, in order to efficiently reach distal neurons, these substrates can be taken up, and distributed through networks of astrocytes connected by GJs, a process modulated by neuronal activity. Astrocytic networks can be morphologically and/or functionally altered in the course of various pathological conditions, raising the intriguing possibility of a direct contribution from these networks to neuronal dysfunction. The present review upgrades the current view of neuroglial metabolic coupling, by including the recently unravelled properties of astroglial metabolic networks and their potential contribution to normal and pathological neuronal activity.

## Introduction

Brain information processing comes with important energetic costs (Harris et al., 2012). Consequently, although representing only 2% of the body mass, the brain consumes 20% of the oxygen and glucose supplied by blood. One of the oldest functions attributed to astrocytes consists in providing metabolic support to neurons. As early as 1886, Camilio Golgi described the strategic position that astrocytes occupy between blood vessels and neurons, and from which the concept of neuroglial metabolic coupling originates. Astrocytes take up glucose, which can then be stored as glycogen or metabolized into lactate, an energy substrate that can be shuttled to neurons and fuel their TCA cycle (Pellerin et al., 2007). Astrocytes can also regulate glucose neuronal supply through the modulation of blood flow via vasodilatation or vasoconstriction of blood vessels, which respectively increases or decreases glucose supply (Attwell et al., 2010). Thus, astrocytes play an important role in the local supply of energy substrates to neurons, via regulation of blood microcirculation as well as glucose uptake and metabolization. However, the molecular mechanisms enabling energy substrates delivery from astrocytes to neurons and the actual role such supply undertakes in sustaining neuronal activity are still matter of intense debates. For decades, the contribution of astrocytes to neuronal metabolic support has mainly been examined at the level of an individual astrocyte as part of the neuro-glia-vascular unit, thus overlooking the extraordinary network properties of astrocytes connected by gap junctions (GJ). However, recent data have unravelled the unique properties of GJ-mediated astroglial networks and their role in neurometabolic coupling. The existing model of astroglial metabolic support to neuronal activity should therefore be upgraded to encompass GJ-mediated astroglial networks, which not only influence local synapses, but also modulate distal neuronal circuits. We here review recent data revealing the main properties of astroglial networks, and describe the current understanding of their contribution to the neurometabolic coupling, at both synaptic and circuit level, in normal and pathological situations. The current review therefore aims at extending the classical model of neuroglial metabolic coupling to the network level.

## Astrocytes have unique metabolic features and form a dynamic network

### Pivotal role of astrocytes in brain energy metabolism

Glucose oxidation in the brain is almost exclusively directed toward fulfilling the high energy cost of synaptic transmission. Enhanced neuronal activity translates into an increased uptake of glucose in the active regions (Belanger et al., 2011). Because very little local energetic reserves are available in the central nervous system (CNS), such activity is highly dependent on the finely regulated supply of glucose from the bloodstream. Astrocytes play a central role in this process. They express glucose transporters at their endfeet, which come into close contact with brain capillaries (Belanger et al., 2011). The astrocyte to neuron lactate shuttle (ANLS) hypothesis states that enhanced neuronal activity at glutamatergic synapses stimulates the uptake of glutamate, which is co-transported with sodium (Na^+^) in astrocytes. Restoration of the Na^+^ gradient by the Na/K ATPase pump consumes ATP, whose levels are recovered by an enhanced uptake and glycolysis of glucose in astrocytes. Glycolysis in astrocytes leads to rapid production of lactate, which is then transported to neurons, thereby fueling their energy needs (Pellerin and Magistretti, 1994). Glutamate is also exported back to neurons in the form of glutamine. Since the first description of the ANLS, the molecular mechanisms subtending the tight cooperation between astrocytes and neurons for glucose oxidation in conditions of enhanced neuronal activity have been further detailed and complexified (Belanger et al., 2011). Notably, it was demonstrated that K^+^ released during neurotransmission is an important additional inducer of astrocyte glycolysis and lactate export (Bittner et al., 2011; Ruminot et al., 2011; Choi et al., 2012).

Another possible fate for glucose in astrocytes is its transformation into glycogen, the major energy reserve of the CNS. Glycogen granules are stored exclusively in astrocytes (Magistretti et al., 1993) and can be mobilized rapidly in conditions of aglycemia to sustain neuronal function (Brown et al., 2005). Most importantly, glycogen, and the lactate derived from its degradation, also play a role in non-pathological conditions, as they have been shown to be required for learning and memory (Suzuki et al., 2011).

Glucose is by far the primary metabolic substrate of the brain, but alternative substrates such as ketone bodies (KB) may also be metabolized, provided their concentrations are sufficiently high relatively to that of glucose. This occurs during the neonatal pre-weaning period, during fasting, or when KB plasma levels are artificially increased by a ketogenic diet (Maalouf et al., 2009; Prins, 2012). KB may also be produced locally by astrocytes as these cells have been shown in culture to synthetize KB from fatty acids (Guzman and Blazquez, 2001). Moreover, in particular conditions, astrocytes may overexpress enzymes involved in KB and fatty acid metabolism and increase their oxidation as alternative substrates to glucose (Escartin et al., 2007).

Overall, astrocytes are perfectly equipped to orchestrate a tight regulation of glucose metabolism in response to neuronal activity. They express a large variety of receptors that allow them to sense and respond to neuronal activity. In addition, they exhibit a complex machinery of transporters and enzymes enabling them to metabolize, store and transfer multiple metabolic substrates. Interestingly, astrocytes are primarily oriented toward glucose uptake and oxidation or storage in the form of glycogen, but they also display a marked metabolic versatility, being able to metabolize efficiently KB, fatty acids and glutamate (Escartin et al., 2007; McKenna, 2007). Importantly, most of the aforementioned metabolites are small enough to transit through GJ, hence conveying the intriguing possibility that these substrates may be shuttled between astrocytes in an activity-dependent manner.

### Widespread and regulated gap junction-mediated networks of astrocytes

**Figure 1:**
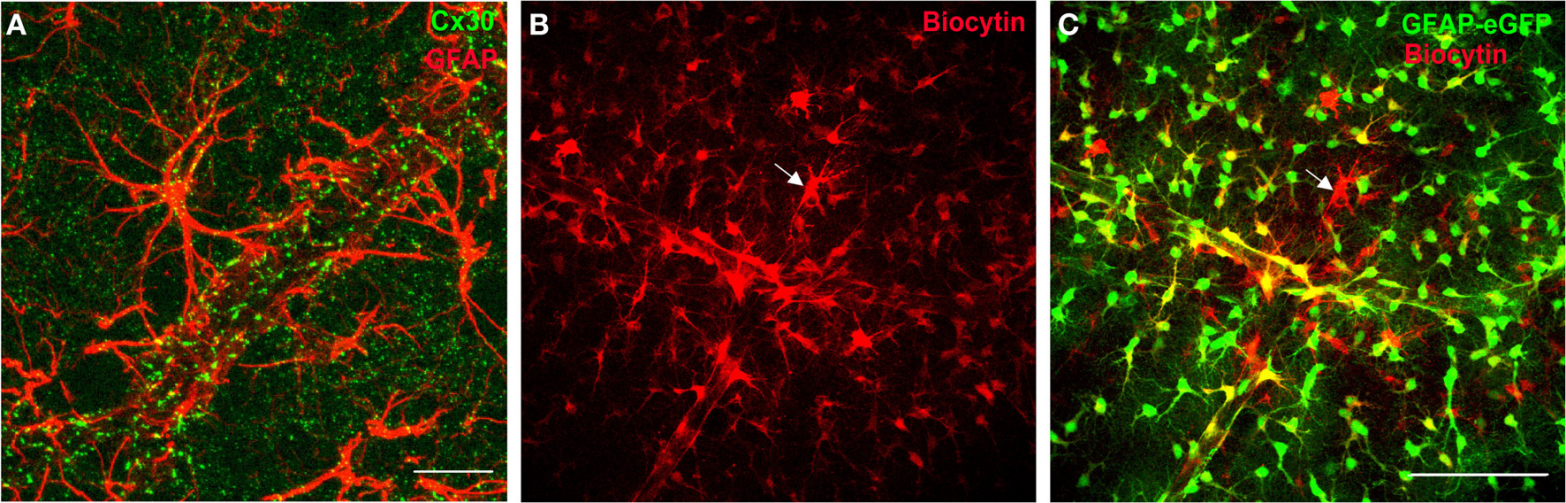
**Connexins define a functional network of astrocytes at the gliovascular interface. (A)** Staining of Cx30, one of the two main GJ protein in astrocytes, in mouse hippocampus co-localizes with astrocyte endfeet, labeled with the glial fibrillary acidic protein marker (GFAP), enwrapping blood vessels. Scale bar, 25 μm. **(B,C)** Functional coupling of perivascular astrocytes in GFAP-eGFP mice visualized by diffusion of biocytin (red, **B**; overlay with GFAP-eGFP, **C**), a tracer permeable to GJ channels, dialyzed for 20 min by whole-cell recording of a perivascular astrocyte (see arrow), revealing an extensive coupling of neighboring astrocytes. Note that some EGFP-positive cells near the dialyzed cell do not stain for biocytin, which indicates the presence of a preferential network of astrocytes. Scale bar, 100 μm. Adapted, with permission, from Giaume et al. (2010) (**A**) and Rouach et al. (2008) (**B**,**C**).

Individual astrocytes play a major role in synaptic physiology by controlling a large number of synapses, in part through gliotransmission, or glutamate and K^+^ uptake (Nedergaard and Verkhratsky, 2012). However, another key property of astrocytes is their extensive network communication via intercellular GJ channels (Figures 1B,C). Astrocytes express indeed high levels of connexins, the proteins forming GJ channels, which are poorly selective channels allowing direct cytoplasmic exchange of a variety of small molecules, with a molecular weight up to 1.5 kDa. These include ions (K^+^, Ca^2+^, Na^+^), second messengers (cAMP, IP3), neurotransmitters (glutamate) or energy metabolites (glucose, lactate) (Giaume et al., 2010). Each GJ channel is formed by the alignment of two hemichannels, the connexons, provided by two neighboring astrocytes, and composed of six transmembrane proteins, the connexins. In astrocytes, the two main connexins expressed are connexin 43, present from embryonic stages to adulthood, and connexin 30, expressed after postnatal day 10 (Nagy et al., 1999). GJ channels mediate the formation of large cellular ensembles reaching millimeter size in different brain regions, encompassing hundreds to thousands of astrocytes (Ball et al., 2007; Giaume et al., 2010).

Astroglial networks display several typical properties. Although GJ mediate an extensive intercellular communication between neighboring cells, these connections can be selective and preferential. Indeed, adjacent astrocytes are not always functionally connected, as assessed by dye coupling experiments (Houades et al., 2006, 2008; Roux et al., 2011) (Figures 1B,C). This may result from heterogeneous expression of connexins in astrocytes, or to short-term regulation of astroglial GJ permeability. Astroglial networks are also finely organized in anatomical and functional compartments, similarly to neuronal networks, as recently shown in different brain areas (Houades et al., 2006, 2008; Roux et al., 2011). For instance, in the hippocampus, astrocytes form extensive and nearly non-overlapping networks in the *stratum radiatum* and *oriens*, respectively, under and above the pyramidal cell layer; this is partly attributable to the non-homogeneous distribution of astrocytes and connexins. More strikingly, in markedly compartmentalized cerebral structures such as the somatosensory cortex or the olfactory bulb, the structural organization of astroglial networks does overlap with the anatomical and functional units of associated neurons. In the somatosensory cortex, astrocytes located between barrels, in the septa, are weakly connected, in contrast to astrocytes located in the barrel, which show significant coupling almost exclusively within a single barrel (Houades et al., 2006). Similarly, in the olfactory bulb, extraglomerular astrocytes show reduced coupling compared to intraglomerular astrocytes, which are mainly coupled within the limits of a single glomerulus (Roux et al., 2011). Confinement of astroglial networks in the reach of a given structural and functional neuronal network may favor specific neuroglial interactions. This might contribute to local and precise processing of neuronal signals, favoring circuit independence by restricting information transfer to distal neurons, belonging to other networks. This region-dependent organization of astroglial networks may also directly influence how distant neurons with high energy needs, within a specific network, are supplied with metabolites taken up by perivascular astrocytes.

## Neurometabolic coupling with networks of astrocytes

### Activity-dependent trafficking of energy metabolites through astroglial networks

**Figure 2:**
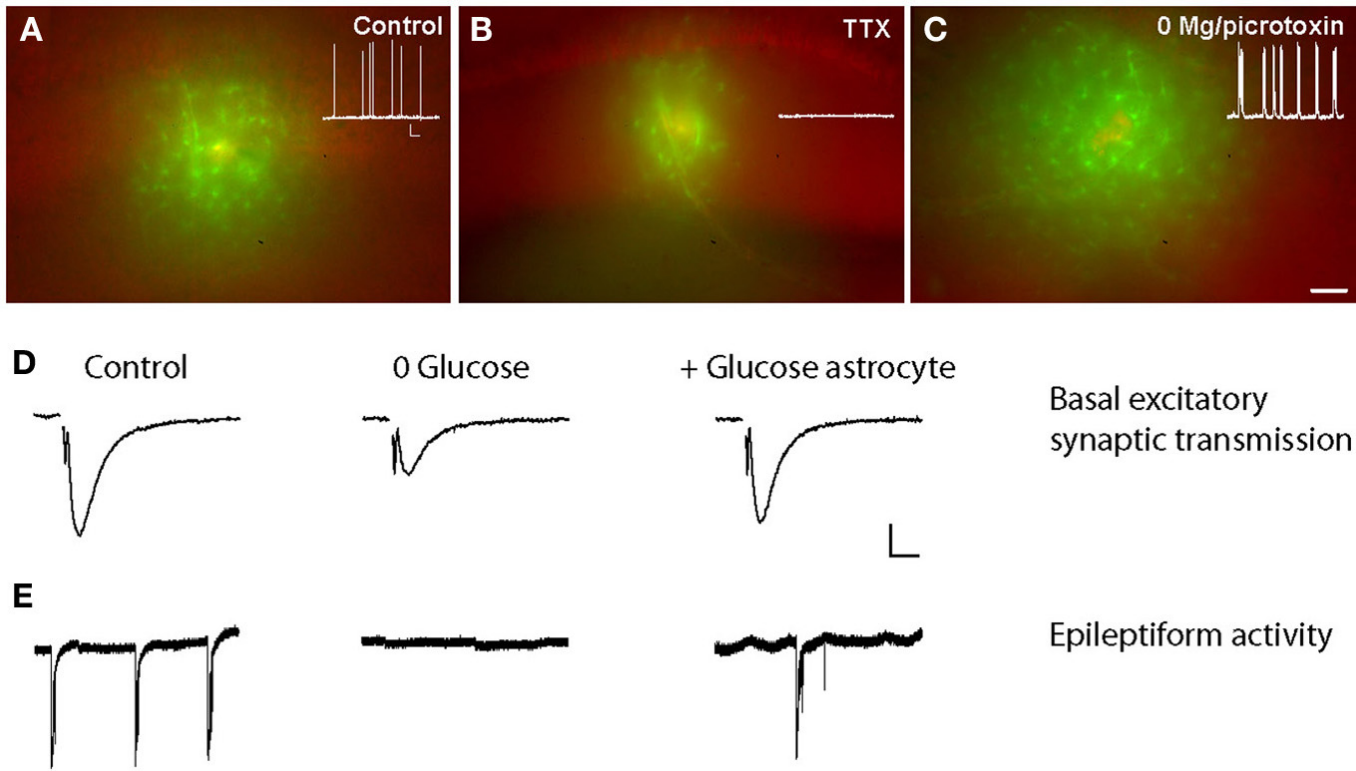
**Activity-dependent glucose trafficking through astroglial networks sustain normal and pathological neuronal activity. (A)** Sample pictures showing that the fluorescent glucose derivative 2-[N-(7-nitrobenz-2-oxa-1,3-diazol-4-yl)amino]-2-deoxyglucose (2-NBDG, green) trafficking in astrocytes is decreased when neuronal activity is inhibited with tetrodotoxin (TTX) **(B)** and increased during epileptiform activity (in 0 Mg^2+^-picrotoxin) **(C)** as compared with control conditions **(A)**. Scale bar, 50 μm. Insets, corresponding spontaneous activity of hippocampal CA1 pyramidal cells recorded in current clamp in control, TTX (0.5 μm, 1–4 h), and 0 Mg^2+^-picrotoxin (100 μm, 1–4 h) conditions. Scale bar, 20 mV, 9 s. **(D,E)** Glucose supply through astrocytic networks sustains basal synaptic transmission and epileptiform activity during exogenous glucose deprivation (EGD). Sample traces of extracellular field potentials recorded in hippocampal slices showing that intracellular glucose (20 mM) delivery to astrocytic networks (+Glucose astrocytes) through the patch pipette inhibits the depression of fEPSP amplitude **(D)** and epileptiform activity **(E)** induced by exogenous glucose deprivation (0 glucose, 30 min) in wild-type mice. Scale bars **(D)** 0.2 mV, 5 ms and **(E)** 0.3 mV, 20 s. Adapted, with permission, from Rouach et al. (2008) **(A–E)**.

A striking feature of Cx43 and Cx30, the two main GJ subunit proteins in astrocytes, is their enrichment in perivascular astrocytic endfeet and delineation of blood vessel walls (Nagy et al., 1999; Simard et al., 2003; Rouach et al., 2008) (Figure 1A). The functional connectivity of perivascular astrocytic networks has thus been hypothesized to contribute to neurometabolic coupling. The first step in demonstrating such hypothesis consisted in visualizing online energy metabolite trafficking through astroglial networks and determining whether it could be subject to activity dependent regulations. This was achievable through the use of fluorescent glucose analogs, such as 2-NBDG (2-[*N*-(7-nitrobenz-2-oxa-1,3-diazol-4-yl)amino]-2 deoxyglucose), infused in single astrocytes lining blood vessels. Diffusion of such molecules revealed that astroglial connexins mediate an activity-dependent glucose distribution through intercellular astroglial networks (Rouach et al., 2008) (Figure 2). The possible contribution of connexin hemichannels in glucose intercellular trafficking was ruled out, because they were shown not to be functional under these physiological conditions (Rouach et al., 2008). Interestingly, glucose trafficking through astroglial networks was found to be specifically regulated, as it occurs within a preferential pathway along interconnected astrocyte endfeet around blood vessels and depends on neuronal activity (Rouach et al., 2008). The molecular mechanism underlying the activity-dependent regulation of astroglial metabolic networks remains, however, unclear. Regulations of astroglial networks have been investigated almost exclusively using passive dye coupling, instead of bioactive molecules. Such approach has enabled to show that the permeability and selectivity of GJ channels control the extent of astroglial network diffusion and are regulated by a variety of endogenous molecules such as ions, peptides, and neurotransmitters, released by various brain cell types and acting on membrane channels and receptors (Giaume et al., 2010). Thus, astroglial networks are functionally plastic and are regulated by neuronal activity, as shown in several brain regions (Fischer and Kettenmann, 1985; Marrero and Orkand, 1996; Rouach et al., 2000, 2002b, 2008; Roux et al., 2011). Although mostly assessed by GJ coupling for passive dyes, a few pathways have been proposed to control activity-dependent astroglial networking. For instance, depolarization mediated by activity-dependent K^+^ release has been suggested to enhance astroglial GJ coupling (Enkvist and McCarthy, 1994; De Pina-Benabou et al., 2001; Roux et al., 2011) through Cx43 phosphorylation by CAMKII (De Pina-Benabou et al., 2001). Glutamate has also been proposed as an important regulator of astroglial GJ permeability, although different effects have been described, depending on the preparation and the receptor subtype involved (Giaume et al., 2010). Glutamate released at hippocampal synapses increases glucose trafficking through astroglial GJs via activation of postsynaptic AMPA receptors, but not astroglial glutamate transporters (Rouach et al., 2008). Interestingly, this activity-dependent regulation of glucose trafficking does not result from a direct action on the permeability of GJ channels, because astroglial GJ coupling for passive tracers is unchanged by glutamate. Thus, glucose trafficking through GJ-mediated astroglial networks may follow a gradient from sites of high concentration, near blood vessels, to sites of low concentration, where active neurons requiring a large consumption reside. This hypothesis is supported by the observation that neuronal stimulation in a given hippocampal layer selectively increases glucose diffusion into this layer via an astroglial network originating from a distant layer (Rouach et al., 2008). Thus, astroglial metabolic networks can be reshaped by local glutamatergic activity from active neurons, which act as a signal for energy demand.

### Metabolic support of synaptic activity

The second step required for probing the role of astroglial networks in neurometabolic coupling was to determine whether intercellular glucose trafficking through astroglial networks could in turn affect synaptic activity. To do so, field excitatory postsynaptic potentials were monitored in the context of exogenous glucose deprivation whilst a remote astrocyte was loaded, via a patch pipette, with glucose or lactate. Exogenous glucose deprivation expectedly induced a slow depression of synaptic transmission, which could be rescued by glucose or lactate supply to a single astrocyte connected to the astroglial network via GJs. Remarkably, such metabolic support was not observed when glucose was administrated to disconnected astrocytes, lacking both Cx30 and Cx43 (Rouach et al., 2008). This body of work therefore suggests that GJ-mediated astroglial networks play a crucial role in the supportive function of astrocytes by providing an activity-dependent intercellular pathway for glucose delivery from blood vessels to neurons. Hence, glutamate increases both glucose uptake and trafficking in astrocytic networks, and is likely to be the key signal for adequate supply of energy metabolites to sites of neuronal demand. The classic model of neurometabolic coupling, in which astrocytes are considered as single entities controlling metabolic substrates supply to neurons, is therefore revisited and now includes GJ-mediated metabolic networks of astrocytes, which provide energy metabolites in a remote yet efficient manner toward sites of active neurotransmission. However up to now, these networks have been characterized *in vitro* and *ex vivo*. Thus, it is still unknown whether extensive astroglial metabolic networks occur *in vivo* and contribute to functional neuroimaging responses observed with *in vivo* techniques such as functional magnetic resonance imaging and positron emission tomography using [^18^F]-fluorodeoxy-glucose.

### Amplification of metabolic responses

One of the key element of the ANLS model is the Na^+^ influx in astrocytes though glutamate transporters, which stimulates glucose uptake. Interestingly, bringing such model to the level of astroglial networks now implies the notion of metabolic response amplification. Indeed, neuronal glutamate has been shown *in vitro* to generate Na^+^-mediated metabolic waves, enabling the coordination of glucose uptake by astrocytes connected by GJ channels (Bernardinelli et al., 2004). This amplification system requires intercellular Ca^2+^ waves to trigger astroglial release of glutamate, which is taken up by glutamate/Na^+^ cotransporters, and results in regenerative intracellular astroglial Na^+^ waves. The recent identification of astroglial intracellular Na^+^ waves *in situ* in the hippocampus (Langer et al., 2012) suggests that this mechanism occurs in physiological conditions. However, generation of Na^+^ waves *in situ* was found to depend on GJ, but not on Ca^2+^ waves. Such observation is reminiscent of the current debate questioning the actual occurrence of astrocytic Ca^2+^ waves in physiological conditions *in situ* and *in vivo*. Nevertheless, a recent study reported that such waves, named glissandi, do occur in physiological conditions *in vivo* in the hippocampus and depend on neuronal activity and GJ channels (Kuga et al., 2011). However, the GJ dependence was demonstrated using carbenoxolone, a non-specific GJ channel blocker targeting as well other ionic channels, which directly regulate neuronal activity (Rouach et al., 2003; Vessey et al., 2004). Whether Na^+^ waves translate into metabolic waves (i.e., waves of enhanced glucose uptake or metabolism) remains to be demonstrated *in situ*. In sum, the generations of metabolic waves through astroglial networks are likely to occur in both, physiological and pathological conditions, and would contribute to neurometabolic coupling over extensive distances.

## Astroglial metabolic networks in pathological conditions

In physiological conditions, GJ-mediated networks of astrocytes play a pivotal role in K^+^ and glutamate buffering, as well as in energy substrate trafficking from blood vessels to distant active neurons. Alterations in GJ permeability are known to exacerbate K^+^ and glutamate dysregulations in pathological conditions such as ischemia (Nakase et al., 2003; Farahani et al., 2005) or epilepsy (Coulter and Eid, 2012; Steinhauser et al., 2012). Intriguingly, the opening of connexin hemichannel is now starting to emerge as a contributing factor to brain diseases (Bennett et al., 2012; Orellana et al., 2012). However, since recent articles have reviewed these two topics in great details (Bennett et al., 2012; Coulter and Eid, 2012; Orellana et al., 2012; Steinhauser et al., 2012), we shall thereafter focus on how changes in GJ-mediated astroglial metabolic networks might influence neuronal dysfunction in several brain diseases such as ischemia, epilepsy or neurodegenerative conditions.

### Which signals through GJs in pathological conditions?

Although the concept of “kiss of death”, whereby apoptotic signals can transit through GJs from a dying cell to a healthy connected cell, was the first put forward (Lin et al., 1998), it remains somewhat controversial. Such process was proposed to account for the amplification of cell death following cerebral ischemia (Frantseva et al., 2002b; Farahani et al., 2005), traumatic brain injury (Frantseva et al., 2002a; Cronin et al., 2008) and infection of astrocytes by HIV (Eugenin and Berman, 2007; Eugenin et al., 2011). Yet the nature of the toxic signals that propagate through the network in each of these specific conditions still remains to be identified to substantiate this hypothesis.

GJ-mediated networks of astrocytes also contribute to the transfer of survival signals such as metabolic substrates, which could supply distal neurons no longer connected to the bloodstream. This might particularly apply to acute local pathological conditions such as ischemia, traumatic brain injury, or infections. In these conditions, local resources are rapidly exhausted and the gap junctional network then represents an invaluable pathway for delivery of glucose metabolites originating from distant sources. For instance, after ischemia, the transfer of energy metabolites from spared regions to the ischemic region might help neurons to overcome local metabolic dearth. However, the shape and extent of astroglial networks being different between brain structures (Theis and Giaume, 2012), the ability of GJ-mediated networks to counteract the deleterious consequences of ischemia may depend on the affected brain region. Interestingly, this might partly explain the discrepancies of the literature regarding the beneficial versus detrimental role of GJs in these diseases.

### How do astroglial metabolic networks function in pathological conditions?

Numerous pathologies have been associated with alterations of astroglial networks and GJ permeability. In particular, changes in connexin expression occur following acute injuries or in the process of chronic diseases, including demyelinating and neurodegenerative diseases (Kielian and Esen, 2004; Giaume et al., 2010; Cotrina and Nedergaard, 2012). Both increases and decreases in astrocyte connexin expression and GJ permeability to passive dyes have been reported, depending on the model, species, and connexin type (Giaume et al., 2010). In neurodegenerative diseases such as Alzheimer's disease (AD), Cx43 expression increases (Koulakoff et al., 2012), resulting in enhanced dye coupling in the cortex of aged transgenic AD mice (Peters et al., 2009). This may contribute to increased astroglial Ca^2+^ waves, as observed in the cortex of transgenic mice with amyloid beta plaques (Kuchibhotla et al., 2009). However, the functional impact of connexin expression alterations on metabolite trafficking through astrocytic networks has never been directly assessed in any of the aforementioned pathologies.

To our knowledge, the contribution of astroglial metabolic networks in the sustaining of neuronal activity in pathological conditions has only been directly assessed in the context of epileptiform activity. In a slice model of acute aberrant discharges (0 Mg^2+^ and picrotoxin), the astroglial network remains functional and the degree of glucose trafficking through the network actually increases, as a consequence of enhanced neuronal activity (Figure 2). Crucially in such context, infusion of glucose or lactate through the astrocytic network, while depriving the acute hippocampal slices from glucose, partially maintained epileptiform activity (Figure 2) (Rouach et al., 2008). This unique study thus suggests that astrocyte metabolic networks are still functional in conditions of acute epileptiform activity and contribute to fuel abnormal neuronal activity.

In other pathological conditions involving, on the contrary, a chronic deficit in energy metabolism, such as neurodegenerative diseases (Lin and Beal, 2006), an increased permeability of GJ-mediated network might represent a beneficial mechanism of resistance, as it may indeed enhance metabolites supply to vulnerable neurons. Once distributed through the network, glucose may be metabolized into lactate to sustain neuronal energy needs, or give rise to reducing equivalents through the pentose phosphate pathway (Allaman et al., 2011; Belanger et al., 2011). Given the involvement of oxidative stress in neuronal demise (Lin and Beal, 2006), such enhancement of the antioxidant machinery would also represent a beneficial aspect.

Metabolic networks of astrocytes therefore combine multiple positive features that might shoulder the compromised neurons in a variety of pathological conditions. Besides neuronal activity, astroglial metabolic networks likely also sustain neuronal survival in many pathological conditions in which GJ channels do still operate (Cotrina et al., 1998; Rouach et al., 2008). Such networks thus represent potent therapeutic targets. However, the current knowledge of astrocytic network functioning in pathological conditions is insufficient and further investigations are required to experimentally assess GJ functions and their impact on disease progression. In addition, targeting GJ metabolic networks demands to identify in these diseases the molecular regulators of connexin expression and GJ permeability.

### The astrocyte network as a therapeutic target

The signaling pathways mediating changes in connexin expression in diseases are still elusive. Pro-inflammatory cytokines such as IL-1β and TNFα decrease the expression of Cx43 and Cx30 *in vitro* (Meme et al., 2006), while the neurotrophic cytokine ciliary neurotrophic factor increases Cx43 expression in reactive astrocytes *in vivo* (Escartin et al., 2006). As a rule, most molecules released in neuroinflammatory conditions impact connexin expression and GJ permeability (Kielian and Esen, 2004). A number of other signals including second messengers, endogenous lipids, and changes in osmolarity or pH also modulate GJ permeability, as assessed using passive dyes (Rouach et al., 2002a). Such changes in permeability are likely to affect GJ network function more transiently than changes in connexin expression, and would hence be a better target to alleviate acute pathological conditions. Besides transcriptional regulation of connexin expression, the molecular cascades governing connexin insertion at the plasma membrane, connexon apposition, and GJ opening probability could also represent relevant targets to modulate network function in brain affections.

## Conclusions and perspectives

Astrocytes have been recognized as major players in neurometabolic coupling for decades. One of the typical features of astrocytes is their massive direct intercellular communication mediated by GJ channels. However, the role of astroglial network organization in their supporting function has only been recently addressed. GJ-connected astrocytes amplify metabolic responses by generating Na^+^-mediated metabolic waves, resulting in coordinated astroglial glucose uptake. In addition, energy substrates, such as glucose and lactate, can traffick in an activity-dependent manner through astroglial networks to sustain distal neuronal activity. Thus, astroglial metabolic networks play a crucial role in neurometabolic coupling, by supplying efficiently and distally energy substrates to active neurons. Given that astroglial metabolic networks are able to provide energy metabolites from distant sources, they likely play important roles in physiological situations associated with increased metabolic demand related to high neuronal activity that exceeds local glucose supply, or pathological conditions with altered substrate availability (such as hypoglycemia, anoxia, ischemia, glucose transporter deficiency).

However, our understanding of the properties and function of astroglial metabolic networks remains insufficient. What might be the advantage of a coordinated glucose uptake and metabolite delivery through astroglial metabolic networks? Theoretically, each astrocyte should be in the reach of a local source of glucose, since the high density of the vascular network within the hippocampus enables every astrocyte to contact a capillary. However, glucose uptake by a single astrocyte in response to energy needs, might be metabolically less proficient than coordinated glucose supply from distal source through astroglial networks in conditions of high neuronal activity. Thus, astroglial metabolic networks may represent an energetically efficient mechanism for glucose delivery to active neurons. In addition, although capillaries are a local source of glucose, the capacity of each astrocyte to take up and deliver glucose to neurons may not be homogeneous within a given brain area. This depends on several factors including the capillary coverage by astrocytic endfeet, the density of glucose transporters in these endfeet, the astroglial metabolic machinery, and the strength of neuroglial interactions (i.e. density of receptors on astrocytes, astrocytic coverage of neurons). These parameters are likely to be heterogeneous among astrocytes (Matyash and Kettenmann, 2010), and thus astroglial GJs may offer a mean to equilibrate neuronal glucose supply in basal or pathological conditions.

Another unknown matter is whether astroglial metabolic networks only operate with excitatory neurons. Astrocytes take up GABA through Na^+^-dependent transporters. Nevertheless, inhibitory activity is associated with lower metabolic demand than excitatory activity (Chatton et al., 2003; Hyder et al., 2006). Therefore, the role played by astroglial metabolic networks at inhibitory synapses remains to be established.

In addition, it is uncertain whether other energy metabolites, such as KBs, known to have a therapeutic effect on epileptic activity, can also traffick through GJs in astrocytes to sustain neuronal activity. The development of fluorescent KBs and other metabolic substrates should help investigating this issue.

Finally, the few studies investigating astroglial metabolic network properties and function have been performed in culture or in brain slices with relatively low temporal and spatial resolution. In addition, they used fluorescent derivatives of glucose, such as 2-NBDG, which are highly bleachable molecules, as well as non-selective blockers of GJ channels, targeting also connexin and pannexin hemichannels, or connexin knockout mouse model chronically disrupting astroglial GJ networks. Hence, the dynamics and moment to moment contribution of astroglial networks to neurotransmission *in vivo* during specific tasks in physiological or pathological situations are still open questions. To solve these issues and better understand brain functions and dysfunctions, the development of novel pharmacological and molecular tools is required, including new stable fluorescent derivatives of metabolite substrates, as well as pharmacological inhibitors and mouse models, timely and selectively targeting the GJ channel function of astroglial connexins. Only then, we shall fully understand the pivotal role played by astroglial networks in neurometabolic coupling.

### Conflict of interest statement

The authors declare that the research was conducted in the absence of any commercial or financial relationships that could be construed as a potential conflict of interest.
